# Porcine circovirus type 2 (PCV2): genetic variation and newly emerging genotypes in China

**DOI:** 10.1186/1743-422X-7-273

**Published:** 2010-10-19

**Authors:** Long J Guo, Yue H Lu, Yan W Wei, Li P Huang, Chang M Liu

**Affiliations:** 1 Division of Swine Infectious Diseases, National Key Laboratory of Veterinary Biotechnology, Harbin Veterinary Research Institute of Chinese Academy of Agricultural Sciences, No.427 Maduan Street, Nangang District, Harbin 150001, P.R. China

## Abstract

**Background:**

Porcine circovirus type 2 (PCV2), the causative agent of postweaning multisystemic wasting syndrome (PMWS), is a serious economic problem for the swine industry in China. In this study, we investigated the genetic variation of PCV2 in China using strains isolated from 2004-2008. Viruses were isolated from samples collected from pigs with multi-systemic lesions and clinical signs of PMWS from different regions of China, and the genomes of these viruses were sequenced. The assembled sequences were used to define the genotypes of these strains; PCR-RFLP methodology was used to distinguish isolates and capture ELISA was used to demonstrate the antigenic changes resulted from ORF2 gene mutation of the isolates.

**Results:**

We identified 19 PCV2 isolates, including four newly emerging PCV2 mutant strains. The 19 isolates were designated into three genotypes (PCV2a, PCV2b and PCV2d). PCV2d represented a novel genotype and a shift from PCV2a to PCV2b as the predominant genotype in China was identified. This is the first report of 1766 nt PCV2 harboring a base deletion at other new different positions. Amino acid sequence analysis identified two novel ORF2 mutations (resulting in ORF2 sequences 705 and 708 nt in length) in three deletion strains (1766 nt) and one strain with a genome 1767 nt in length. Finding of two amino acids elongation of the ORF2-encoded Cap protein is firstly observed among PCV2 strains all over the world. The isolates were distinguished into different genotypes by PCR-RFLP methodology and antigenic changes were present in Cap protein of mutation isolates by capture ELISA.

**Conclusions:**

The results of this study provide evidence that PCV2 is undergoing constant genetic variation and that the predominant strain in China as well as the antigenic situation has changed in recent years. Furthermore, the PCR-RFLP method presented here may be useful for the differential identification of PCV2 strains in future studies.

## Background

Porcine circovirus type 2 (PCV2) is the causative agent of postweaning multisystemic wasting syndrome (PMWS). This disease was first confirmed in Canada in 1997, and was then subsequently identified in pigs in the USA, France, Japan, Korea and other countries [1,2]. In recent years, PMWS has become a serious economic problem for the swine industry in China. PCV2 is a member of the genus *Circovirus*, of the family *Circoviridae*, the smallest non-enveloped, single-stranded, circular DNA viruses that replicate autonomously in mammalian cells. The viral DNA of PCV2, encapsulated by a single viral protein, is a single-stranded negative sense circularized molecule of 1767-1768 nucleotides (nt) [3-5]. Its genome contains two major open reading frames (ORFs), ORF1 which encodes two replication-associated proteins (Rep and Rep") and ORF2 which encodes a viral capsid protein (Cap) involved in the host immune response [6]. Besides PMWS, PCV2 infection has been linked with porcine dermatitis and nephropathy syndrome (PDNS), porcine respiratory disease complex (PRDC), reproductive failure, granulomatous enteritis, necrotizing lymphadenitis, exudative epidermitis and congenital tremors [7-14]. The pathogenesis of PMWS caused by PCV2 remains to be fully elucidated. However, PCV2 pathogenicity is thought to be mediated by the interaction of this virus with the host immune system. The immune response to PCV2 infection and the subsequent immunological suppression of the host have been subjects of investigation in recent years [15-18].

A recent report into the genetic variation of PCV2 identified nine different genotypes in clinical tissue specimens collected from pigs in different regions of China between 2001 and 2003, by PCR and restriction fragment length polymorphism (RFLP) analysis of the ORF2 region. The results indicated that mutations existed in the genome of the predominant PCV2 strain in pigs in China [19]. Previous investigations by Fenaux *et al*. [20] suggested that the genome sequence of PCV2 strains differed depending on the area of isolation. Recently, Dupont *et al*. [21] showed that the genome sequence of PCV2 was not directly correlated to its pathogenicity, and they reported a strain with a genome of 1766 nt. Since 2003, variation has been reported in the predominant genotypes of PCV2 worldwide, and genotype PCV2b was thought to be the most prevalent form showing enhanced pathogenicity. Mutations were also reported in PCV2 strains in Korea [22]. In 2005, Knell *et al*. reported elongation in the Cap protein as a result of a single nucleotide deletion in the genome [23]. Olvera *et al*. [24] also reported a mutation within the ORF2 gene, which resulted in elongation of the Cap protein.

Recently, to address the current scientific confusion on genotype names, the EU consortium on porcine circovirus diseases http://www.pcvd.net proposed a unified nomenclature for PCV2 genotypes. The consortium proposed naming the three PCV2 genotypes: PCV2a, PCV2b and PCV2c. Using this system, ORF2 sequences of PCV2 are assigned to different genotypes when the genetic distance between them is at least 0.035 [25].

PCV2 mutations are monitored and reported worldwide, however, there have been few reports [19,26] of the genetic variation of PCV2 in China. The aim of this study was to determine the genetic variation of PCV2 in China using strains isolated from 2004-2008 from PMWS-affected herds (cases). This information may provide a valuable insight into the molecular epidemiology and pathogenic mechanisms of PCV2 infection, as well as strategies for the prevention and control of this virus. We also established a PCR- and RFLP-based method to differentiate different genetic isolates in the field.

## Materials and methods

### Sample collection

A total of 90 samples were collected from unthrifty pigs (ranging from 1 to 3 months of age) with multi-systemic lesions and clinical signs of PMWS in different regions of China from 2004-2008. The geographical origin, year of sample collection, age, clinical history, organs/tissue type and the number of samples are summarized in Table 1. The samples were used for DNA extraction and PCR. A PCV2 reference strain (LG) was also maintained in our laboratory and used in this study [27].

**Table 1:** Identification and genotype results of nineteen PCV2 isolates from different geographic distribution materials with clinical history in China.

Isolates name	Isolate region	Age(weeks)	Clinical history	Isolate tissue	Isolate time	Genotype	GenBank No	Genome length(nt)
JF	Jilin	6	PMWS, respiratory signs	Inguinal lymph node	2008	PCV2b	HM038022	1767
SY	Jilin	4	PMWS, PNDS	Tonsil	2008	PCV2b	HM038028	1767
HN	Hunan	9	PMWS	Inguinal lymph node	2006	PCV2b	HM038021	1767
JS	Jiangsu	6	PMWS	Inguinal lymph node	2005	PCV2b	HM038024	1767
AS	Liaoning	5	PMWS	Serum	2005	PCV2b	HM038016	1767
SH	Shanghai	7	PMWS	Spleen	2006	PCV2b	HM038027	1767
WF	Shandong	7	PMWS, diarrhea	Serum	2007	PCV2b	HM038029	1767
CF	Neimenggu	11	PMWS	Inguinal lymph node	2005	PCV2b	HM038019	1767
MZL	Neimenggu	6	PMWS, respiratory signs	Spleen	2008	PCV2b	HM038025	1767
JMS	Heilongjiang	7	PMWS, respiratory signs	Tonsil	2007	PCV2b	HM038023	1767
QTH	Heilongjiang	10	PMWS, PNDS	kidney	2007	PCV2b	HM038026	1767
HL	Heilongjiang	8	PMWS	Inguinal lymph node	2004	PCV2b	HM038020	1767
YJ	Jilin	3	PMWS	Liver	2008	PCV2b	HM038032	1766
BY	Heilongjiang	8	PMWS	Tonsil	2005	PCV2b	HM038018	1767
BDH	Heilongjiang	7	PMWS, Abortion	Serum	2008	PCV2d	HM038017	1767
MDJ	Heilongjiang	5	PMWS, diarrhea	Inguinal lymph node	2007	PCV2d	HM038031	1766
AH	Anhui	5	PMWS	Serum	2008	PCV2d	HM038030	1766
CL	Jilin	9	PMWS, respiratory signs	lung	2007	PCV2a	HM038033	1768
LG	Jilin	12	PMWS	Inguinal lymph node	2008	PCV2a	HM038034	1768

### Virus isolation

Ninety samples were analyzed by PCR for the presence of PCV2. To isolate PCV2, positive samples were freeze-thawed three times, fragmented and centrifuged. Filtered supernatants were inoculated onto porcine kidney PK-15 cells, free of PCV1 contamination. The PK-15 cells were maintained at 37°C with 5% CO_2 _in minimum essential medium (MEM) (Gibco BRL, USA) and 5% heat-inactivated fetal bovine serum (FBS) (Gibco BRL). PCV2 was isolated from the culture supernatants and the isolates were then analyzed by PCR and an immunoperoxidase monolayer assay (IPMA) to confirm the presence of PCV2.

### PCR amplification of genomic DNA

The purified PCV2 genomic DNA was PCR amplified using the primer pairs: PCV2-F (920-946 nt; 5"-ATCCACGGAGGAAGGGGGCCAGTT-3") and PCV2-R (925-901 nt; 5"-GTGGATTGTTCTGTAGCATTCTTCCA-3"). PCR reactions (25 μl) contained 5 μl of KOD DNA polymerase reaction buffer (TOYOBO Biotechnology Co. Ltd., Japan), 0.1 mM of each deoxynucleoside triphosphate, 0.4 μM of each primer, 1 μl (1 unit) of KOD-Plus-Ver.2 high fidelity DNA polymerase (TOYOBO Biotechnology Co. Ltd.) and 100 ng of purified DNA. PCR was performed on a thermocycler under the following conditions: 5 min at 94°C, followed by 35 cycles of 30 s at 94°C, 30 s at 61°C and 2 min at 72°C, and a final step of 10 min at 72°C.

### Cloning of the viral genome

To isolate PCV2 DNA, viral isolates were freeze-thawed three times and then centrifuged. The supernatant was then used as a template for PCR amplification of the PCV2 geneome as previously described. Amplicon products were purified using the QIAquick PCR purification kit (Qiagen, Germany), following the manufacturer's instructions. Prior to cloning of the purified DNA fragment, an A tail was added to the 3" terminus of the amplicon by incubation of 12.5 μl of Premix Taq Hot start version mixture (TaKaRa Biotechnology Co. Ltd., China) with 12.5 μl of purified PCR product at 72°C for 30 min. The amplicon with an A tail was then purified using the QIAquick PCR purification kit, cloned into the pMD18 T vector system (TaKaRa) and transformed into *Escherichia coli *TOP10 competent cells. The resulting colonies were screened according to the manufacturers' instructions. Positive colonies were detected using the PCR protocol described above, except that the first denaturation step was performed at 94°C for 10 min. Plasmid DNA was extracted using the Axygen Plasmid Miniprep Kit (AXYGEN Biotechnology Co. Ltd., China) according the manufacturers' instructions and recombinant plasmids were identified by restriction enzyme analysis with *Sal *Ⅰ/*Xba *Ⅰ.

### DNA sequencing of the cloned PCV2 genomic DNA and sequence analysis

Plasmids from ten different colonies per strain were selected for sequencing at a commercial facility (Sangon Biotechnology Co., Ltd., China), and both strands of the insert were sequenced at least twice, using the M13 universal primers. The sequences of the DNA fragments were then assembled using DNAMAN software (Version 5.2.2, Lynnon Biosoft, 1994). Then, multiple alignments of nucleotide and deduced amino acid sequences were performed using ClustalW within the DNASTAR software (version 7.0). MEGA version 4 was used with Kimura's two parameter correction method to align the sequences, estimate nucleotide distances and diversities (mean nucleotide distances between genotypes and within genotypes) and assess the phylogenetic relationships by the neighbor-joining method using 1000 bootstrap replicates and the 19 strains isolated in this study, together with 154 PCV2 strains deposited in the GenBank database.

### PCR-RFLP analysis of virus isolates

PCR was performed as described above and PCR products were digested with 1 U of the restriction enzymes *Acc*Ⅰand *Fba*Ⅰ (TaKaRa), in a volume of 30 μl for 4 h at 37°C. The digestion products were then separated by electrophoresis on 1% agarose gels.

### Reactivity of the 19 isolates with monoclonal antibody 1D2 by capture ELISA

Mice were immunized with 250 μg of purified PCV2 recombinant Cap (PCV2-rCap) protein, prepared by the baculovirus expression system and monoclonal antibody (mAb) 1D2, was prepared using the lymphocyte hybridoma technique. The mAb ascites titers reached 1:2,048,000, the mAb isotype was IgG2a and the mAb light chain was κ chain for mAb 1D2. Western blot analysis showed that mAb 1D2 was not able to react with native PCV2-Cap protein, but it was able to react with PCV2 via IPMA and antigen capture ELISA, and could be differentiated with PCV1 under the same conditions. The mAb 1D2 was shown by a neutralization test to neutralize PCV2 (data not shown). For the antigen capture ELISA, 96-well microtiter plates were coated with swine anti-PCV2 positive sera (1:100 dilution) in 0.1 M carbonate buffer (pH 9.6) at 4°C overnight, and were then blocked with 5% skimmed milk for 3 h. After blocking, the plates were washed three times with PBST (PBS with 0.1% Tween 20). In the binding assay, plates were incubated with the culture supernatant of the 19 isolates (10^4.5^TCID_50_/ml), respectively, at 37°C for 1 h, followed by three washes with PBST. Bound isolates were detected with horseradish peroxidase (HRP)-coupled 1D2 mAb (the neutralizing mAb of mouse anti-PCV2 Cap). Then, 100 μl of 2,2'-azino-bis(3-ethylbenzthiazoline-6-sulphonic acid (ABTS) was added and after 30 min the reaction was stopped with 2 M H_2_SO_4 _and the absorbance at 405 nm was measured using an ELISA plate reader (Bio-Rad, Hercules, CA, USA).

## Results

### Virus isolation and identification

**Figure 1:**
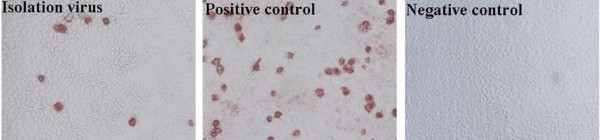
**Identification of 19 isolated viruses by IPMA**. Each of the 19 isolates reacted with swine anti-PCV2 positive sera. Strain PCV2/G was included as a positive control and PK15 cells were used as a negative control.

**Figure 2:**
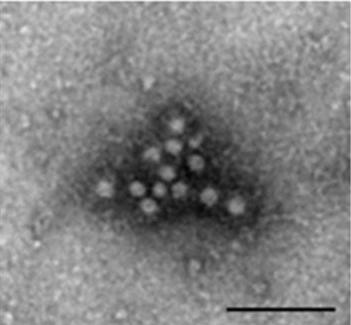
**Immune electron micrograph of negatively stained rescued PCV2 particles (bar = 100 nm)**. The diameter of particles was about 17 nm corresponding to the size reported formerly.

Of the 90 samples, 42 samples (46.7%) were PCV2 PCR positive (data not shown). Nineteen PCV2 strains were isolated from the PCV2 PCR positive samples (Table 1) and they were confirmed as PCV2 by PCR, an antigen-based IPMA (Figure 1) and Immune electron (Figure 2).

### Cloning of the genomic DNA from the virus isolates

The full-length PCV2 PCR products amplified from the DNA extracted from the virus-infected cells were inserted into the pMD18-T vector. Plasmid DNA was then extracted from transformed cells and digested with *Sal*I and *Xba*I. Two bands of the expected sizes were obtained for each strain, indicating that the genomes of the 19 PCV2 strains had been successfully cloned into the vectors.

### DNA sequencing of the cloned viral genomes

Ten positive recombinant plasmids were selected for each isolated strain, and the DNA sequencing data obtained using the M13 universal primers revealed that there was 95.3-100% similarity at the nucleotide level among the genomes of the 19 strains. The strains were categorized into three types groups depending on the length of their genome: three (15.8%) strains had a genome of 1766 nt, 14 (73.7%) strains had a genome of 1767 nt and two (10.5%) strains had a genome 1768 nt in length (Table 1). These 19 sequences were submitted to the GenBank database and assigned accession numbers HM038016 to HM038034. The sequencing results indicated that the predominant PCV2 strain in China had a genome of 1767 nt. Three additional strains with a genome of 1766 nt (with one deleted base) were isolated. There was no clinical history associated with these PCV2 sequences and the pathogenicity associated with these strains warrants further investigation.

### Designation of genotypes

The genotypes of the genomic sequences of the 19 isolated strains were designated according to the method of Grau *et al*. [25]. Two strains belonged to the PCV2a genotype, 14 strains belonged to the PCV2b genotype and three strains belonged to the PCV2d genotype, accounting for 10.5%, 73.7% and 15.8% of the isolated strains, respectively (Table 1).

### Mutation analysis of the PCV2 genome sequences

**Figure 3:**
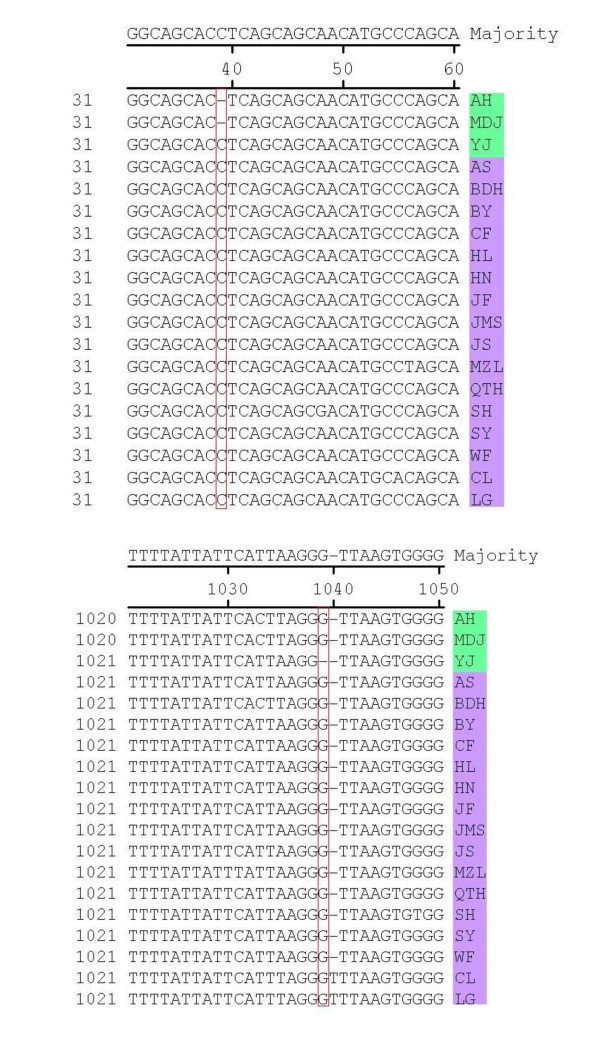
**Sequence alignment of a fragment of the genomic sequence of the 19 PCV2 isolates**. Alignments were performed using the DNA Star 6.0 software. The red boxes indicate the position of three mutations: a one base deletion at position 39 (AH and MDJ) and 1039 (YJ). An insertion mutation of one base (T) at position 1040 is also shown.

Sequence comparisons between the 19 strains revealed that three strains had a single base deletion and therefore represented a new mutant strain of PCV2 with a genome 1766 nt in length. More detailed sequence analysis using the DNASTAR software showed that two strains (AH and MDJ), with genomes 1766 nt in length, had a single base deletion (C) at position 39, while the other strain (YJ) had a single base deletion (G) at position 1039. In addition, two strains (LG and CL), with genomes 1768 nt in length, were found to possess a base insertion (T) at position 1040 (Figure 3).

### Amino acid sequence analysis of the putative ORF2-encoded Cap protein

**Figure 4:**
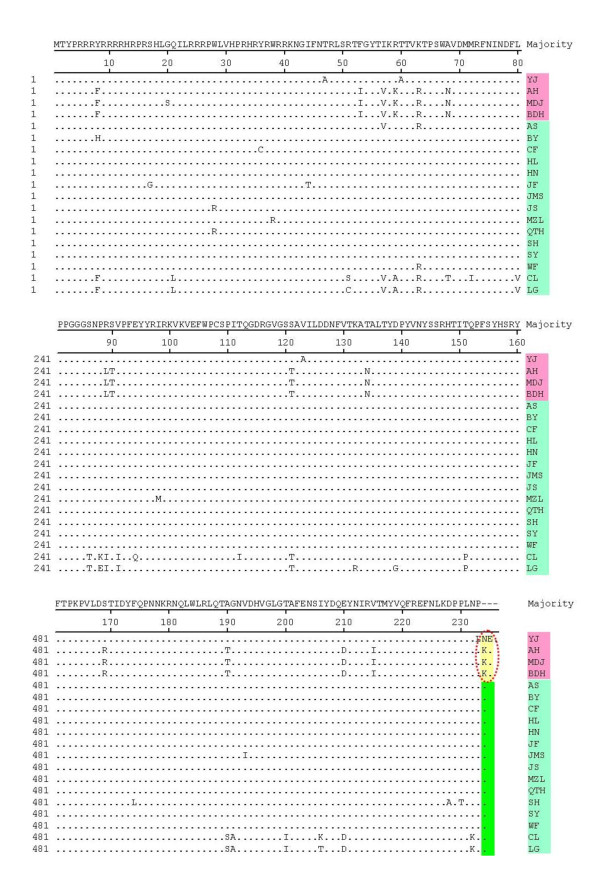
**Sequence alignment of the deduced amino acid (aa) sequences of the capsid proteins (encoded by ORF2) of the 19 PCV2 isolates**. Mutations at the C terminus of the capsid protein in four isolates are shown, resulting in elongation of the ORF by one lysine residue (AH, MDJ and BDH) or by an asparagine and a glutamic acid residue (YJ), compared with the wild-type sequence (green box).

**Figure 5:**
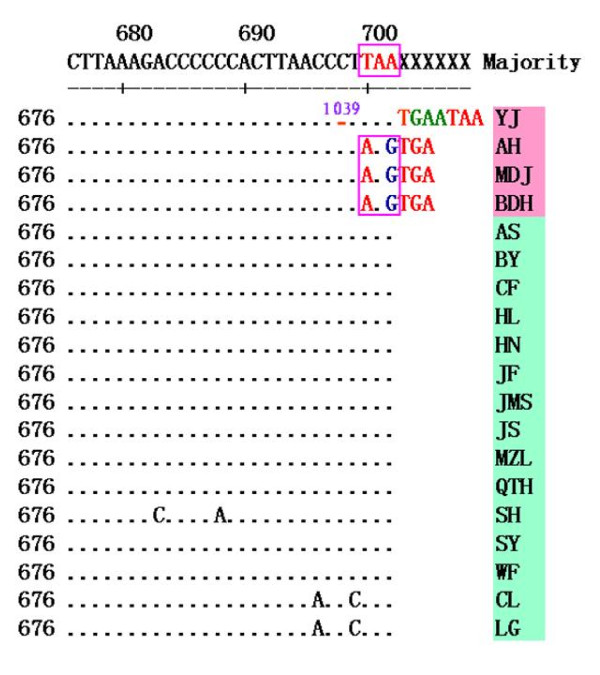
**Alignment of the ORF2 gene sequences (702/705/708 bp) encoding the capsid protein in the 19 isolates, using the ClustalW program within the DNA Star 6.0 software**. Mutations in the stop codon in four isolates (YJ, AH, MDJ and BDH) resulted in elongation of the ORF by one codon, and a one base deletion in isolate YJ at position 1039 led to a two-codon elongation of the ORF.

The 19 ORF2-encoded Cap protein sequences were relatively conserved, although a few mutations existed (Figure 4). Important mutations located in the C terminus of the Cap protein are indicated by the red circle in Figure 4. Strain YJ showed an elongation of two amino acids (Asn and Glu), and strains AH, BDH and MDJ showed an elongation of one amino acid (Lys) compared with the PCV2a isolations. Analysis of the ORF2 gene in the 19 strains indicated a one-base deletion at position 1039 in the genome of strain YJ, resulting in an ORF2 gene of 708 nt. While in strains AH, BDH and MDJ, a stop codon mutation in the ORF2 resulted in an ORF2 gene of 705 nt (Figure 5). ORF2 gene was elongated by one or two codons due to the deletion and mutation mentioned above.

### Construction of the phylogenetic tree

**Figure 6:**
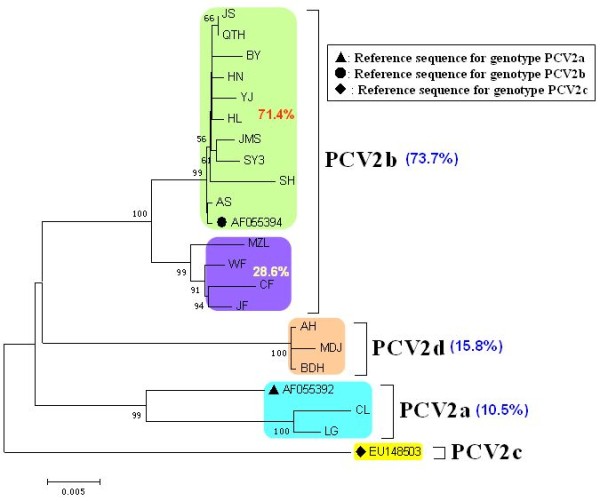
**Phylogenetic analysis of the 19 PCV2 isolates**. The phylogenetic tree was constructed using MEGA 4 software after alignment of the full-length sequences. The PCV2 genomes were mainly assigned to three genotypes (PCV2a, PCV2b and PCV2d). PCV2b was subdivided into two clusters and PCV2d represented a newly emerging genotype in China. The percentage of strains in each genotype or cluster is indicated at the corresponding positions. The results showed that the PCV2b genotype of PCV2 is predominant in China, which was compatible with reports of a shift from PCV2a to PCV2b.

**Figure 7:**
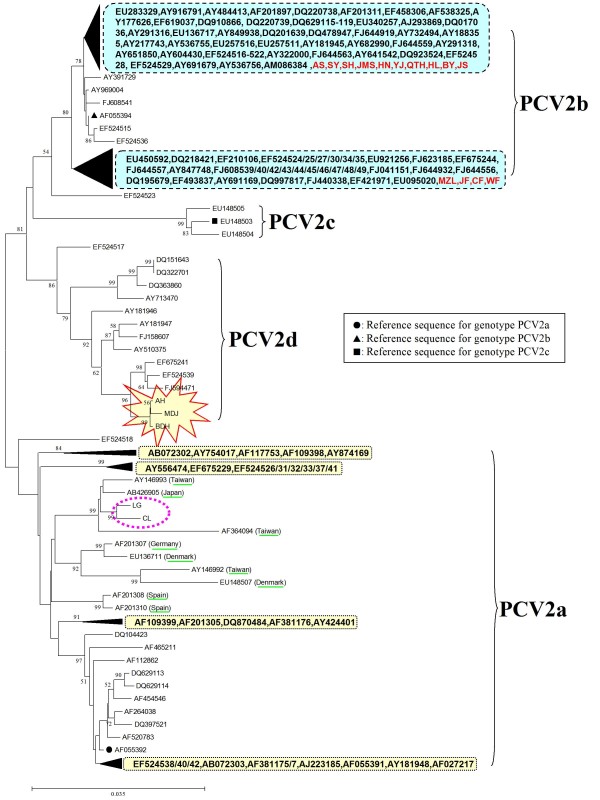
**Phylogenetic trees based on 175 PCV2 full-length sequences, including the 19 isolates from this study and 156 strains submitted to GenBank**. The accession numbers for these strains are indicated and some branches in the tree are collapsed in the form of black triangles, followed by their corresponding accession numbers. The tree was constructed using a neighbor-joining algorithm with the MEGA 4 software. Three main genotypes (PCV2a, PCV2b and PCV2c), along with a newly emerging genotype PCV2d, were identified by multiple alignments, and PCV2b was subdivided into two clusters. Genotype PCV2b was predominant among strains in China. Three PCV2d isolates from this study form a single branch, marked by a red box. Two PCV2a isolates from this study (marked by a pink circle) were separated from the other Chinese PCV2a isolates and were instead located at a mixed branch with strains from Taiwan, Japan and Europe (underlined in green).

Sequence analysis was carried out on the genomes of the 19 PCV2 strains using the MEGA 4 software, and a phylogenetic tree was constructed. Analysis of the phylogenetic tree indicated that the sequences could be divided into three genotypes (PCV2a, PCV2b and PCV2d) and the PCV2b genotype strains were subdivided into two clusters, which accounted for 71.4% and 28.6% of the PCV2b genotype strains respectively (Figure 6). The phylogenetic tree constructed using the 19 sequenced strains, together with 156 sequences previously deposited in GenBank (including sequences reported in Zhou *et al*., [26] and Wang *et al*., [28] as well as reference sequences for different PCV2 genotypes found at http://www.pcvd.net/) is shown in Figure 7. The 175 sequences included in this analysis can also be divided into three genotypes (PCV2a, PCV2b and PCV2c), together with another genotype designated PCV2d in this study. Genotype PCV2d viruses are reported not only in this study but also in Wang *et al*. demonstrating that this new genotype is prevalent in China [28]. Of the 19 strains isolated in this study, the majority belonged to genotype PCV2b (73.7%), indicating that genotype PCV2b viruses are the predominant genotype circulating in China. The genomic distances between the genotypes ranged from 0.0411 to 0.0637. Within genotypes, the average genomic distances ranged from 0.0100 (PCV2c) to 0.0293 (PCV2a). The PCV2d isolates identified in this study with an amino acid elongation in the C terminus of the Cap protein, formed a separate branch from other Chinese isolates of the PCV2d genotype. Two PCV2a isolates identified in this study were separated from other Chinese PCV2a isolates, being located in a mixed branch with isolates from Taiwan, Japan and Europe. This indicated that a new PCV2 cluster was present within the PCV2a genotypes (Figure 7).

### Characterization of the isolates by PCR-RFLP

**Figure 8:**
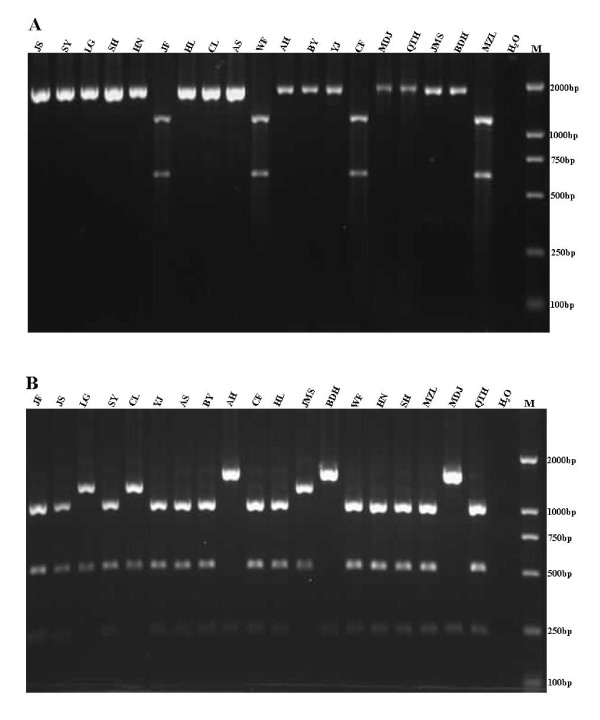
**PCR-RFLP profiles of the amplified PCV2 genomic DNA after digestion with restriction endonucleases *Fba *I (A) and *Acc *I (B)**. Isolate names are indicated above the corresponding lanes. Panel A shows the *Fba *I digest of the PCR amplification fragments and restriction fragments are evident at 1165 and 602 bp for the strains in one cluster of genotype PCV2b, the DNA of other strains remains undigested. Panel B shows the *Acc *I digest of the PCR amplification fragments and restriction fragments are evident at 1257 and 511 bp (PCV2a); 1532 and 235 bp (PCV2d); and 1021 511 and 235 bp (the other clusters in genotype PCV2b, except the JMS strain).

Genomic PCR amplification products from the 19 isolated PCV2 strains were characterized by digestion with restriction enzymes *Fba*Ⅰand *Acc*Ⅰ, and analysis of the resulting electrophoretic patterns. Four strains (JF, WF, CF and MZL) forming a single cluster in the genotype PCV2b isolates were differentiated from the remaining strains by digestion with the restriction enzyme *Fba*Ⅰ(Figure 8A), while strains from genotypes PCV2a and PCV2d, and the remaining PCV2b strains, were identified by digestion with *Acc*Ⅰ(Figure 8B). These results correlated with the phylogenetic tree analysis, indicating that analysis via PCR-RFLP using two restriction enzymes, could be an effective method for differentiation of PCV2 isolates in the field.

### Identification of antigenic differences between the 19 isolates

**Figure 9:**
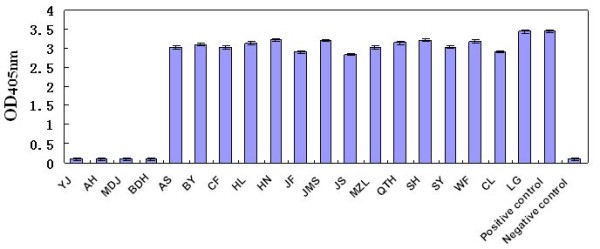
**Reactivity of the 19 isolates with mAb 1D2**. All isolates showed strong reactivity with mAb 1D2 except the YJ isolate (PCV2b) and the PCV2d genotype isolates (AH, MDJ and BDH), indicating that viruses of the PCV2d genotype are not recognized by mAb 1D2 and that the ORF2 mutation in strains of this genotype results in antigenic changes in the PCV2 Cap protein.

Antigen capture ELISA using mAb 1D2 showed that all isolates had strong reactivity with this mAb, except for isolate YJ (PCV2b), and isolates AH, MDJ and BDH (PCV2d). These results indicated that viruses of the PCV2d genotype were not recognized by mAb 1D2, and that mutation of the ORF2 gene resulted in antigenic changes within the PCV2 Cap protein (Figure 9).

## Discussion

Disease caused by PCV2 infection has increased in severity in China in recent years, resulting in increased morbidity and mortality in swine and significant economic losses in the swine industry. Genetic variation among PCV2 isolates has been reported by researchers worldwide in recent years [17,19,21,24]. A novel PCV-like agent was first isolated from pig sera in China [29], but few studies have since been performed on PCV2 genetic variation in China. Studies are therefore required which investigate PCV2 variation in pigs in China and assess the most prevalent PCV2 genotypes, to facilitate prevention and control of PCV2-induced disease.

Of the 19 isolates analyzed in this study, two strains of the PCV2a genotype were separated from other Chinese PCV2a genotypes on the phylogenetic tree, and were located in a new mixed branch along with strains from Taiwan, Japan and Europe. The occurrence of these two distinct PCV2a genotype strains indicated that either foreign PCV2 viruses had entered China via international trade/travel, or that these strains had resulted from the same evolutionary events occurring in different regions of the world simultaneously. In this study, we also identified the existence of a novel genotype, PCV2d, in China among 12 formerly isolated strains and three new isolates (this study). These 15 isolates all carried the same mutated ORF2 gene of 705 bp in length, except for two of the formerly isolated strains (GenBank no. DQ151643 and DQ322701), and further investigations are required to determine the potential relationship between this mutation and pathogenicity of the viruses.

Previous investigations have shown that the genomes of PCV2 viruses are usually 1767 or 1768 nt in length, however, strains (GenBank no. EU148507 and EU257516) with a genome of 1766 nt have also been reported [21,30]. In this study, a phylogenetic tree constructed from the 19 isolates identified in this study and the 156 strains submitted to GenBank (GenBank no. are shown in Figure 7) indicated that the gene deletion strain (GenBank no. EU148507) isolated in Denmark belonged to genotype PCV2a and it was genetically similar to the genotype PCV2a strains, suggesting that it may have evolved from a strain harboring a genome of 1768 nt. The gene deletion sequence (GenBank no. EU257516) of the 1766 nt genome strain previously reported by Shang *et al*. [30], belonged to genotype PCV2b and was genetically similar to the genotype PCV2b strains, suggesting that it may be evolved from the 1767 nt genome strain (Figure 7). By sequence alignments and analysis, it was found that the positions of base deletions differed among strains with genomes of 1766 nt. A deletion (C) was found at position 39 in the genomes of two strains (1766 nt) that were isolated in this study (AH and MDJ), compared with a reference strain with a genome of 1767 nt, while a deletion (G) was found at position 1039 in the genome of another strain (YJ). In contrast, a deletion (G) was found at position 1060 in the genome of the strain reported by Shang *et al *[30]. (GenBank no. EU257516), and two deletions (T) were found at positions 1002 and 1003 in the genome of the Denmark strain (GenBank no. EU148507). So, in our study, we isolated 3 strains with 1766 nt in genomic length harboring a base deletion at different positions from other reports [21,30] in genome, and other reports [19,28,31] only reported common strains with a genome of 1767 nt or 1768 nt. Although the sites of deletion mutations varied among strains, these results suggested that mutations occur frequently in the epidemic strains of PCV2. Further studies are now required to investigate whether these kinds of gene mutations are associated with viral pathogenicity.

The Cap protein, encoded by ORF2, is the major structural protein of PCV2 and the major protective antigen. It is relatively conserved, but a few mutations have been identified in this gene in recent years, and were also identified in this study. An elongation of one or two amino acids was found in the C terminus of the Cap protein due to alteration of the ORF2 gene in three 1766 nt genome strains (AH, MDJ and YJ) and one strain with a genome of 1767 nt (BDH). Knell *et al*. [23] had previously reported that mutations could occur in the ORF2 gene since a deletion (T) was found at position 1042 in the 1767-nt genome of a strain (GenBank no. AY713470), which led to a one amino acid elongation of lysine in the C terminus of ORF2-coded Cap protein. Olvera *et al*. [24] also reported elongation by one lysine (K) residue of the C terminus of the Cap protein in a mutation in the stop codon of the ORF2. Similarly, in this study, an elongation of one lysine (K) residue was identified in the C terminus of the Cap protein in three strains (AH, MDJ and BDH) due to a mutation in the stop codon of the ORF2. Besides, in the study, we first reported a 1766 nt strain (YJ) with a ORF2 of 708 nt all over the world. The ORF2 gene of the strain was mutated since a deletion was found at position 1039 of the genome, which led to the elongation of two amino acids (N and E) at the C terminus of the ORF2-encoded Cap protein. This finding of two amino acids elongation of the ORF2-encoded Cap protein is firstly observed among PCV2 strains all over the world (the rescued PCV2 based on the genome sequence of YJ strain has also obtained by infectious molecular clone, data not show). The two amino acids elongation finding also proved that PCV2 underwent variations frequently. Besides, strain YJ, BDH, AH and MDJ showed no reactivity with the neutralizing mAb against PCV2 Cap protein, indicating changes in the antigenic epitopes expressed on the Cap protein (Figure 9). The relationship between deletion mutations and changes in the antigenicity, pathogenicity and virulence of viruses, warrants further investigation.

A PCR-RFLP method was established in this study for the genotyping of PCV2 epidemic strains which could be used to identify genotypes and clusters of strains. Former report about PCR-RFLP for PCV2 only got the differences between different strains, but can't distinguish the strains into different genotypes. So, in this study, the method used only two enzymes (*Fba*Ⅰ and *Acc*Ⅰ) to identify the different genotypes of PCV2 in the field, which seems more simple and useful than the method reported by Wen *et al *[19]. The enzymes *Fba*Ⅰ and *Acc*Ⅰ were used and the results correlated with the results of the phylogenetic tree (with the exception of strain JMS), providing a simple and effective method for genotyping and identifying epidemic strains of PCV2. The appearance of strain JMS also suggested that PCV2 strains are acquiring ongoing mutations. In summary, we have identified four different PCV2 genotypes circulating in China. PCV2d represented a novel genotype and we report a shift from PCV2a to PCV2b as the predominant genotype. We also constructed a simple PCR-RFLP based method to genotype and identify PCV2 strains. Besides, antigenic differences were identified between isolates by mAb 1D2.

## Conclusion

Taken together, we identified 19 PCV2 isolates, including four newly emerging PCV2 mutant strains. The 19 isolates were designated into three genotypes (PCV2a, PCV2b and PCV2d). PCV2d represented a novel genotype and a shift from PCV2a to PCV2b as the predominant genotype in China was identified. This is the first report of PCV2 harboring a base deletion at other new different positions in 1766 nt strains. In this study, finding of two amino acids elongation of the ORF2-encoded Cap protein is firstly observed among PCV2 strains all over the world. Amino acid sequence analysis identified two novel ORF2 mutations (resulting in ORF2 sequences 705 and 708 nt in length) in three (1766 nt) deletion strains and one strain with a genome 1767 nt in length. In addition, mutation of the ORF2 gene resulted in antigenic changes within the PCV2 Cap protein by using the mAb 1D2 in capture ELISA assay. This indicated that different genotypes as well as newly emerging PCV2 were present with antigenic difference of Cap protein, that is the main structural protein of PCV2.

## Competing interests

The authors declare that they have no competing interests.

## Authors' contributions

LJG organized the whole process, took part in all the experiments and wrote the manuscript. CML designed the whole project. YHL participated in the clinical materials collection; YWW made great contribution to the virus isolation; LPH carried out the cell culture. All authors read and approved the final manuscript.
